# Weight development in children with obesity without treatment: A Danish cohort study with long‐term follow‐up

**DOI:** 10.1111/ijpo.70001

**Published:** 2025-02-04

**Authors:** Rasmus Møller Jørgensen, Jens Meldgaard Bruun, Mette Fogh, Iris Iglesia Altaba, Luis A. Moreno, Henrik Støvring, Jane Nautrup Østergaard

**Affiliations:** ^1^ Steno Diabetes Center Aarhus Aarhus University Hospital Aarhus Denmark; ^2^ Department of Clinical Medicine Aarhus University Aarhus Denmark; ^3^ Danish National Center for Obesity Aarhus Denmark; ^4^ Growth, Exercise, Nutrition and Development (GENUD) Research Group University of Zaragoza, Instituto Agroalimentario de Aragón (IA2), Instituto De Investigación Sanitaria Aragón (IIS Aragón) Zaragoza Spain; ^5^ Primary Care Interventions to Prevent Maternal and Child Chronic Diseases of Perinatal and Developmental Origin Network (RICORS) Instituto de Salud Carlos III Madrid Spain; ^6^ Faculty of Health Sciences Universidad Internacional de La Rioja Logroño La Rioja Spain; ^7^ CIBER Fisiopatología de la Obesidad y Nutrición Instituto de Salud Carlos III Madrid Spain; ^8^ Department of Biomedicine Aarhus University Aarhus Denmark; ^9^ Clinical Pharmacology, Pharmacy and Environmental Medicine University of Southern Denmark Odense Denmark

## Abstract

**Introduction:**

Limited insight exists into the weight development in children with obesity not receiving obesity treatment.

**Methods:**

This cohort study included 467 Danish children aged 5–10 years with obesity (iso‐BMI >30 kg/m^2^) not receiving treatment. Data from mandatory health check‐ups on school‐children's height and weight (converted to BMI *z*‐scores) were merged with the Danish National Registries. A multivariable logistic regression weighted for the duration of follow‐up was used to estimate odds ratios (OR) for normalization of BMI (iso‐BMI 18.5–25 kg/m^2^) and obesity remission (iso‐BMI 18.5–30 kg/m^2^).

**Results:**

During a median follow‐up of more than 6 years, 7.9% of the children normalized their BMI, while 45.4% obtained obesity remission. BMI *z*‐score at inclusion acted as a strong inverse predictor for normalizing BMI (OR 0.14 per one‐unit SD, CI: 0.03–0.53) and for obesity remission (OR 0.17 per one‐unit SD, CI: 0.08–0.37). No other significant predictors were observed in the weighted multivariable models.

**Conclusion:**

Higher BMI *z*‐scores inversely predict normalizing BMI and achieving obesity remission in untreated children. Given that many children naturally achieve obesity remission or weight normalization, resources should focus on understanding barriers of obesity maintenance and to develop effective strategies for those who do not experience improvement.

**Abbreviations**
BMI: body mass index
CI: confidence intervals
IOTF: International Obesity Task Force
ISCED: International Standard Classification of Education
Q_1_;Q_3_
: lower and upper quartile
SES: socioeconomic status
SD: standard deviation

## 1 INTRODUCTION

The obesity prevalence in children and adolescents has quadrupled worldwide since 1990 and the NCD Risk Factor Collaboration estimates that approximately 160 million children and adolescents were living with obesity in 2022.^1^ Childhood obesity has been associated with negative impact on mental health,^2^, ^3^ but also with the development of somatic complications later in life.^4^, ^5^, ^6^

Childhood and adolescent obesity strongly predicts obesity later in life with a fivefold increased likelihood of obesity in adulthood compared to their peers without obesity.^7^ Factors associated with the risk of developing obesity in childhood have been thoroughly examined,^8^, ^9^, ^10^, ^11^ but only limited insight exists into the weight development among children with obesity not enrolled in lifestyle interventions (4–11 years). In studies not adjusting for potential obesity treatment or lifestyle interventions, up to 10% of children with obesity have been reported to obtain a body mass index (BMI) within a normal range (iso‐BMI 18.5–25 kg/m^2^), and up to 29% have entered obesity remission (iso‐BMI 18.5–30 kg/m^2^).^12^, ^13^, ^14^ The risk of persistent obesity seems to be associated with the degree of obesity,^9^, ^15^ high birthweight,^13^ and low socioeconomic status (SES)^12^, ^14^; however, these associations have not been consistently reported.^13^ Age plays an important role for changes in weight status in children with obesity.^7^, ^15^, ^16^, ^17^ Children aged 5–6 years have a higher chance of obesity remission as compared to older children.^17^ Another critical time period for children seems to be around 12–14 years of age, where children with severe obesity (iso‐BMI ≥35) gain weight with faster rates^16^; however, factors such as being a girl, not being part of some ethnic minorities, and having better interpersonal skills also predict a greater chance of remission.^18^, ^19^

By identifying children with the potential to normalize BMI or obtain obesity remission without treatment, healthcare professionals working with in this population may be able to differentiate the treatment, and resources could be allocated to those with a higher risk of weight gain or persistence of obesity. This could eventually reduce unnecessary stigmatization, parental concern, and money spent on interventions.

The national registers at Statistics Denmark provide an ideal setting to investigate potential predictors for reversal or remission of obesity in children not enrolled in obesity treatment. Danish children are assessed regularly by school health nurses and general practitioners during childhood. This includes mandatory health check‐ups during school hours performed by school health nurses when the child is 5–6, 9–10, and 14–15 years of age.^20^

The primary aim of this study was to identify potential predictors for obtaining a BMI within a normal range (iso‐BMI 18.5–25 kg/m^2^) and obesity remission (iso‐BMI 18.5–30 kg/m^2^) in Danish children aged 5–10 years living with obesity and not receiving lifestyle intervention. As a secondary aim, we investigated potential predictors for being among the children with the highest increase in BMI *z*‐score (being in the highest quintile) during follow‐up.

## 2 METHODS

### 2.1 Study design and setting

This observational cohort study included children aged 5–10 with obesity followed for minimum of 3 years, who were not invited to participate in a family‐centered lifestyle intervention and living in Aarhus Municipality, Denmark, between 1 January 2010 and 17 March 2018. The end of follow‐up was determined from the date of last data extraction by Aarhus Municipality. The children were identified and followed by using the mandatory health check‐ups at school and then merged with information on child and family characteristics from the Danish national registers.^21^, ^22^

### 2.2 Ethical approval

The study was conducted in accordance with the updated ethical standards of the 1964 Helsinki Declaration,^23^ approved by the Central Jutland Regional Local Committee on Health Ethics (record no. 1‐45‐70‐27‐20) and by the Danish Data Protection Agency. The study has also been reported to clinicaltrials.gov (protocol id: NCT06077266).

### 2.3 Study population

We identified 2337 children and adolescents with obesity in TM‐Sund during the study period. Obesity was defined by the International Obesity Task Force (IOTF) guidelines (iso‐BMI ≥30 kg/m^2^, corresponding to BMI ≥30 kg/m^2^ as in adults).^24^, ^25^ TM‐Sund is a data‐capturing tool used by school health nurses in Aarhus Municipality to record measures of height and weight obtained at the health check‐ups. The inclusion visit was defined as the first observation, where the child could be classified as having obesity.

We included children who had no record of being offered treatment in the lifestyle intervention by Aarhus Municipality. The treatment offered by Aarhus Municipality is a one‐year municipality‐driven family‐centered behavioural modifying lifestyle intervention, which is managed by specialized school health nurses. In a recently published paper, Jorgensen et al. reported, that children accepting the intervention offered by Aarhus Municipality were younger and a higher proportion were girls as compared to the children not invited to participate. A detailed description of the intervention can be found in Jorgensen et al.^26^

**FIGURE 1:**
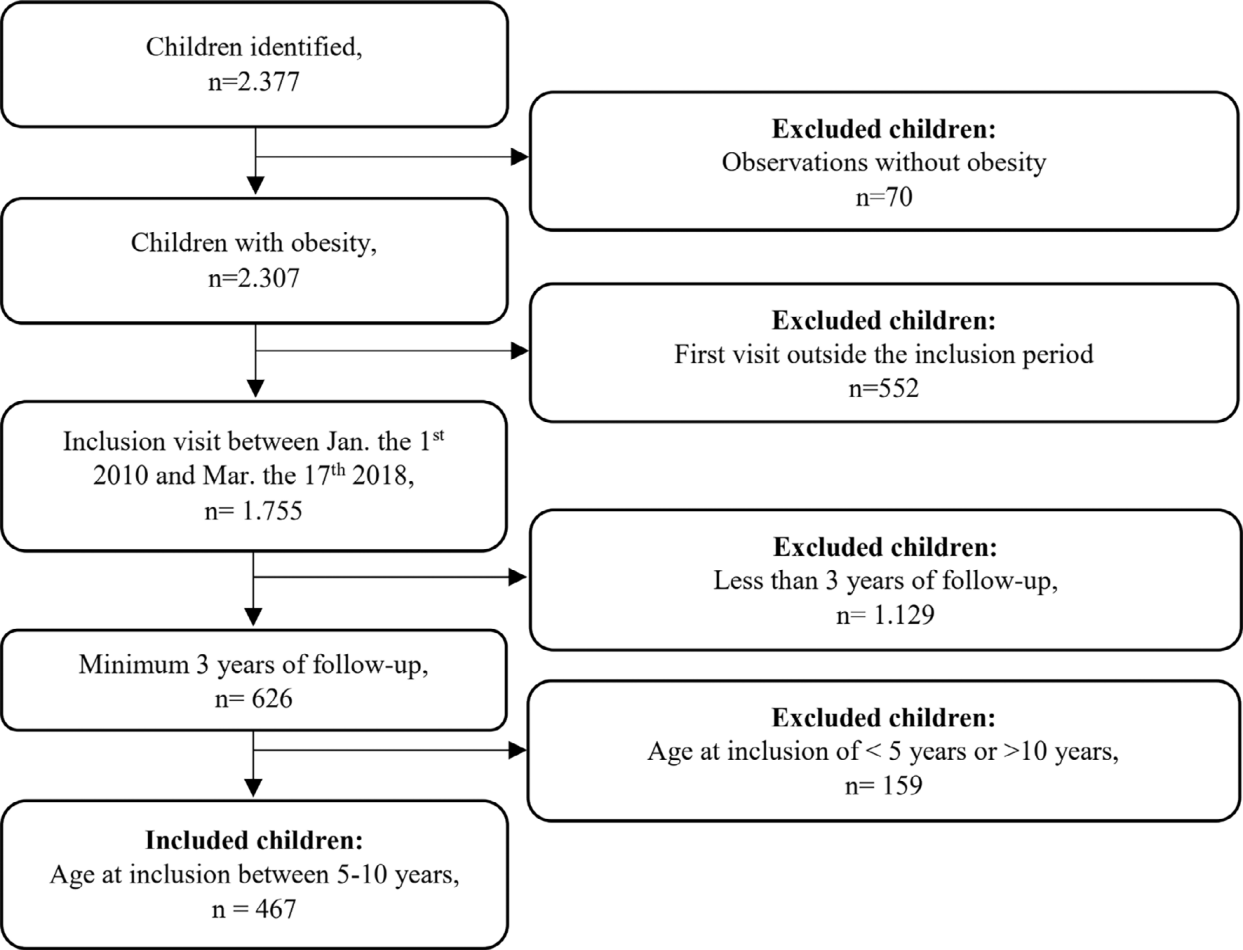
Flowcharts for inclusion and exclusion of children with obesity aged 5–10 living followed for a minimum of 3 years living in Aarhus Municipality, Denmark between 1 January 2010 and 17 March 2018, and not invited to participate in the lifestyle intervention.

Inclusion criteria were obesity,^24^, ^25^ age 5–10 years, and a minimum follow‐up of 3 years. In total, we included 467 children, while 1910 children were excluded from further analyses (Figure 1).

Children were lost to follow‐up if they moved out of the municipality. However, the study design made it impossible to quantify the number of children lost to follow‐up this way.

### 2.4 Study variables

Height and weight were recorded by specialized school health nurses by using a fixed stadiometer and with the child wearing light clothing, empty pockets, and no shoes. For each observation, we categorized the child as normal BMI (iso‐BMI 18.5–25 kg/m^2^ for age and sex) or having overweight or obesity (iso‐BMI ≥25 kg/m^2^ for age and sex) and as obesity remission (iso‐BMI < 25 kg/m^2^ for age and sex) or, persistence of obesity (iso‐BMI ≥30 kg/m^2^ for age and sex).^24^ A corresponding BMI *z*‐score (standard deviation (SD)) adjusted for age and sex was calculated for each observation by using a validated Danish reference tool.^27^ The change in BMI *z*‐score was calculated by subtracting the BMI *z*‐score at inclusion from the BMI *z*‐score at the last observation.

The primary outcome in this study was a BMI within a normal range and obesity remission at the last obtained observation. As our secondary outcome, we identified children with the largest increase in BMI *z*‐score at the last obtained observation, by dichotomizing the children as being in the highest quintile (above the 80th percentile) of change in BMI *z*‐score or being among the remaining children (below the 80th percentile).

Equivalised household income and highest completed household education were used as proxies for SES.^28^, ^29^, ^30^, ^31^ The highest completed parental education was recoded into three categories by the International Standard Classification of Education (ISCED): primary education, ISCED 1–2 (Short), high school, vocational education short, and short‐ medium‐cycle higher, ISCED 3–5 (Middle) and Tertiary education on a university degree or equivalent, ISCED 6–8 (Long).^28^, ^29^ The Equivalised Household Income is adjusted for taxes, alimony, and number of family members and reported in Danish Kroner (DKK).^30^, ^31^ The children were classified as either being part of a family with two adults or not. Immigration status was defined as having Danish origin or having an immigrant background (first or second generation) by using The Danish Population Register.^32^ Children were classified as having mental disorders if a psychiatric diagnosis had been recorded at a hospital admission or outpatient clinic,^33^, ^34^ while a family history of mental disorder was classified if a parent with a psychiatric diagnosis was recorded at a hospital.

### 2.5 Statistical analysis

We tabulated sex, median age, mean BMI *z*‐score, family type, equivalised household income, highest completed household education, immigration status, psychiatric diagnosis, and family history of mental illness at inclusion to describe the study population. We observed missing data for the variables family type (*n* = 4 (0.9%)), highest completed parental of education (*n* = 6 (1.3%)), equivalised household income (*n* = 66 (14%)), and immigration status (*n* = 4 (0.9%)). These were kept as missing in the tables and in the logistic regression. As comparison between the groups at inclusion, we used the Student's unpaired *t*‐test to compare normal distributed data, the Wilcoxon Rank Sum test to compare non‐normal distributed data, and the Fisher's exact test to compare categorical variables (data not shown, but mentioned in the text).

To describe the change of weight classes in a Danish context, we calculated the proportion of children with a BMI within a normal range and children obtaining a remission of obesity. We performed the analyses in several steps. First, we used both a univariable and a multivariable logistic regression to estimate the odds ratios (OR) for normalizing BMI as compared to having overweight or obesity, obesity remission as compared to having persisting obesity, and the highest increase in BMI *z*‐score (being above compared to being below the 80th percentile), to adjust for differences in the length of follow‐up, the multivariable models were weighted by the ratio of follow‐up of each individual relative to the maximum follow‐up observed in the cohort. The multivariable logistic regression analyses were adjusted for no more than one co‐variable per 10 observations of the least common outcome and the co‐variables were prioritized in the following order; sex, age, BMI z‐score, highest completed parental of education, immigration status, psychiatric diagnosis, and family history of mental illness and family type. This order was pre‐defined by reviewing the existing literature and by expert discussions.

We reported estimates with 95% confidence intervals (CI) and used 5% as the significance level. Analyses were performed in Stata 18 College Station, TX: StataCorp LLC.^35^

## 3 RESULTS

### 3.1 Normalization of BMI

In total, 37 (7.9%) children changed weight status from having obesity to a BMI within a normal range during follow‐up, while 430 (92.1%) children were classified as having either overweight or obesity at the last observation. The median follow‐up for the children normalizing BMI was longer compared to children who did not (7.5 years (lower and upper quartile (Q_1_;Q_3_): 6.0; 8.3) and 6.2 years (Q_1_;Q_3_: 4.8;7.5), respectively).

The only significant difference at inclusion was the mean BMI z‐score, which was lower in the group normalizing BMI (2.8 SD, 0.3) compared to the group with overweight or obesity at the end of follow‐up (3.1 SD, 0.5) (Table 1).

**TABLE 1:** Characteristics of 467 children with obesity aged 5–10 with a minimum of 3 years of follow‐up living in Aarhus Municipality, 2010–2018 and not receiving a lifestyle intervention.

		Normalization of BMI		Obesity remission	
	Overall	Normal weight at end of follow‐up	Overweight/obesity at end of follow‐up	Remission of obesity at end of follow‐up	Persistent obesity at end of follow‐up
*N*	467	37	430	212	255
Sex, *n* (%)					
Boys	228 (48.8%)	16 (43.2%)	212 (49.3%)	91 (42.9%)	137 (53.7%)
Girls	239 (51.2%)	21 (56.8%)	218 (50.7%)	121 (57.1%)	118 (46.3%)
Age at inclusion, mean (SD)	8.0 (1.3)	7.9 (1.3)	8.0 (1.4)	7.9 (1.3)	8.2 (1.4)
BMI‐*z* score (inclusion), mean (SD)	3.0 (0.5)	2.8 (0.3)	3.1 (0.5)	2.9 (0.4)	3.2 (0.6)
Family type, *n* (%)					
Not two‐parents family	160 (34.6%)	11 (29.7%)	149 (35.0%)	75 (35.7%)	85 (33.6%)
Two‐parents family	303 (65.4%)	26 (70.3%)	277 (65.0%)	135 (64.3%)	168 (66.4%)
Equivalised household income (DKK), mean (SD)^a^	161 397 (1.0)	187 067 (1.1)	159 482 (1.0)	166 020 (1.0)	157 690 (1.0)
Highest completed education, *n* (%)					
Short	97 (21.0%)	5 (13.9%)	92 (21.6%)	35 (16.7%)	62 (24.6%)
Middle	243 (52.7%)	22 (61.1%)	221 (52.0%)	111 (53.1%)	132 (52.4%)
Long	121 (26.2%)	9 (25.0%)	112 (26.4%)	63 (30.1%)	58 (23.0%)
Immigrants status, *n* (%)					
Danish	231 (49.9%)	21 (56.8%)	210 (49.3%)	119 (56.7%)	112 (44.3%)
Immigrants	232 (50.1%)	16 (43.2%)	216 (50.7%)	91 (43.3%)	141 (55.7%)
Psyciatric diagnosis, child, *n* (%)					
No	448 (95.9%)	>32 (>86.4%)	413 (96.0%)	203 (95.8%)	245 (96.1%)
Yes	19 (4.1%)	<5 (<13.5%)	17 (4.0%)	9 (4.2%)	10 (3.9%)
Family history of mental illness, *n* (%)					
No	320 (68.5%)	27 (73.0%)	293 (68.1%)	152 (71.7%)	168 (65.9%)
Yes	147 (31.5%)	10 (27.0%)	137 (31.9%)	60 (28.3%)	87 (34.1%)

*Note*: The children have been stratified as having normal weight (iso‐BMI 18.5–25 kg/m^2^) or overweight/obesity (iso‐BMI ≥25 kg/m^2^) as well as remission of obesity (iso‐BMI 18.5–30 kg/m^2^) or persistent obesity (iso‐BMI ≥30 kg/m^2^).

^a^ Log‐back transformed.

**FIGURE 2:**
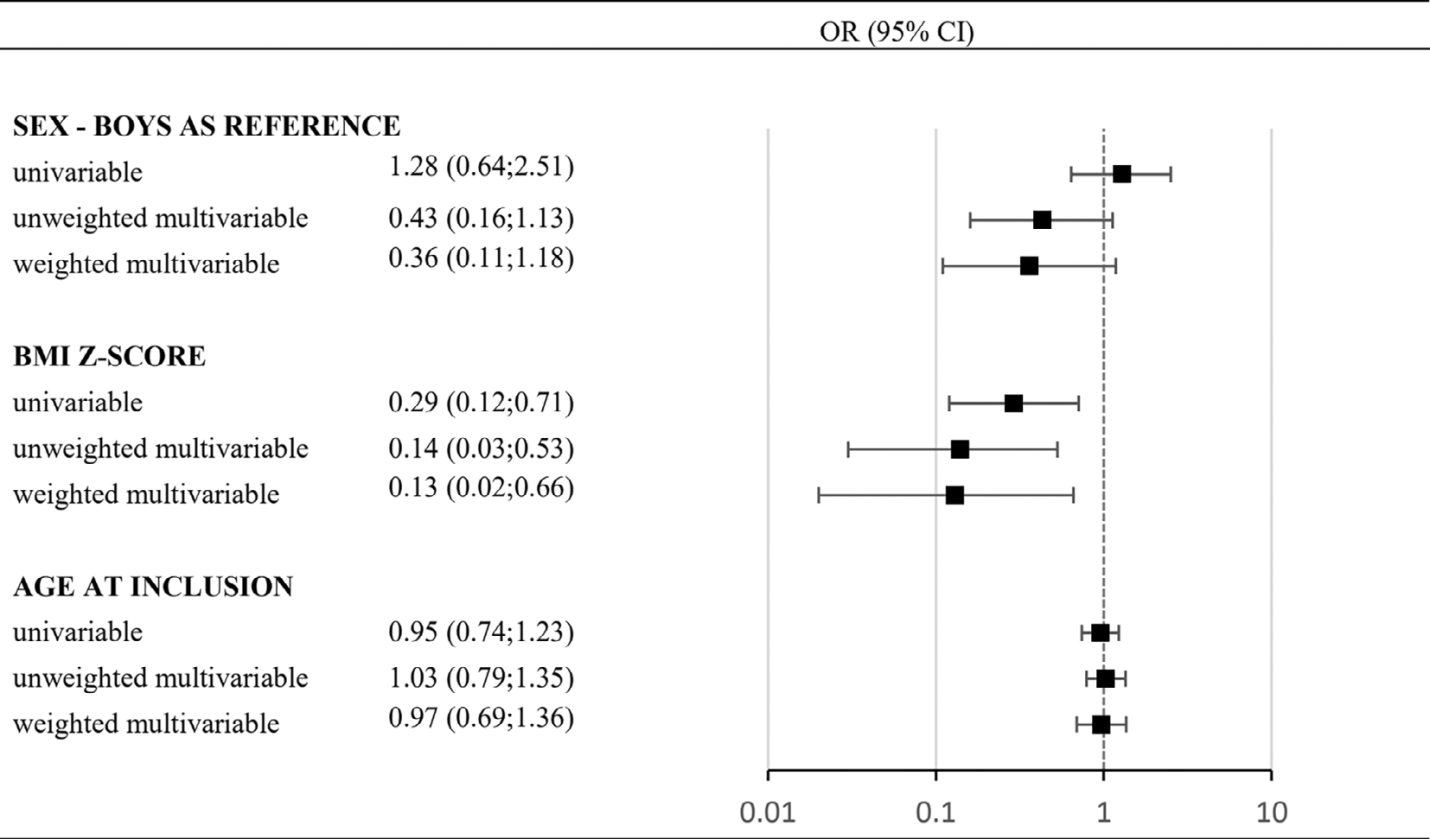
Logistic regression models, including 467 children aged 5–10 years living with obesity and a minimum of 3 years of follow‐up not receiving a lifestyle intervention, present the OR of normalizing weight at the end of follow‐up with having overweight or persistent obesity as the reference group. For each co‐variable, we present univariable, unweighted multivariable, and weighted multivariable OR. The included co‐variables are sex (boys as reference), BMI *z*‐score (per unit increase in SD), and age (per unit increase in a year). The weighted multivariable model was weighted by the ratio of follow‐up of each individual relative to the maximum follow‐up observed in the cohort.

An inverse association between BMI *z*‐score and the odds of normalizing BMI (OR 0.14 per unit SD, CI: 0.03–0.53) was observed in the weighted multivariable model, while a non‐significant inverse tendency was observed for the girls compared to boys (OR 0.36, CI: 0.11–1.18). Results including sex, BMI *z*‐score, and age were similar in the corresponding univariable and unweighted multivariable models (Figure 2). Additionally, in the univariable model, there were no significant associations between normalizing BMI and family type, highest completed household education, immigration status, psychiatric diagnosis, and family history of mental illness (data not shown).

### 3.2 Remission of obesity

Remission of obesity, that is, change to normal weight or overweight was observed in 212 children (45.4%), while 255 children (54.6%) were still classified as having obesity at the end of follow‐up. We did not observe differences in follow‐up time between the groups, 6.4 years (SD: 1.8) for the children obtaining obesity remission and 6.1 (SD: 1.7) for the persistent obesity group, respectively. At the time of inclusion in the descriptive comparison, the children obtaining obesity remission were younger, more likely to be girls than boys, had a lower BMI *z*‐score, and were more likely to have Danish origin as compared to the children in the persistent obesity group (Table 1).

We observed an inverse association between BMI *z*‐score and the odds for obtaining obesity remission (OR 0.17 per one‐unit SD, CI: 0.08;0.37) in the weighted multivariable model and this was similar in the corresponding univariable and unweighted multivariable models.

**FIGURE 3:**
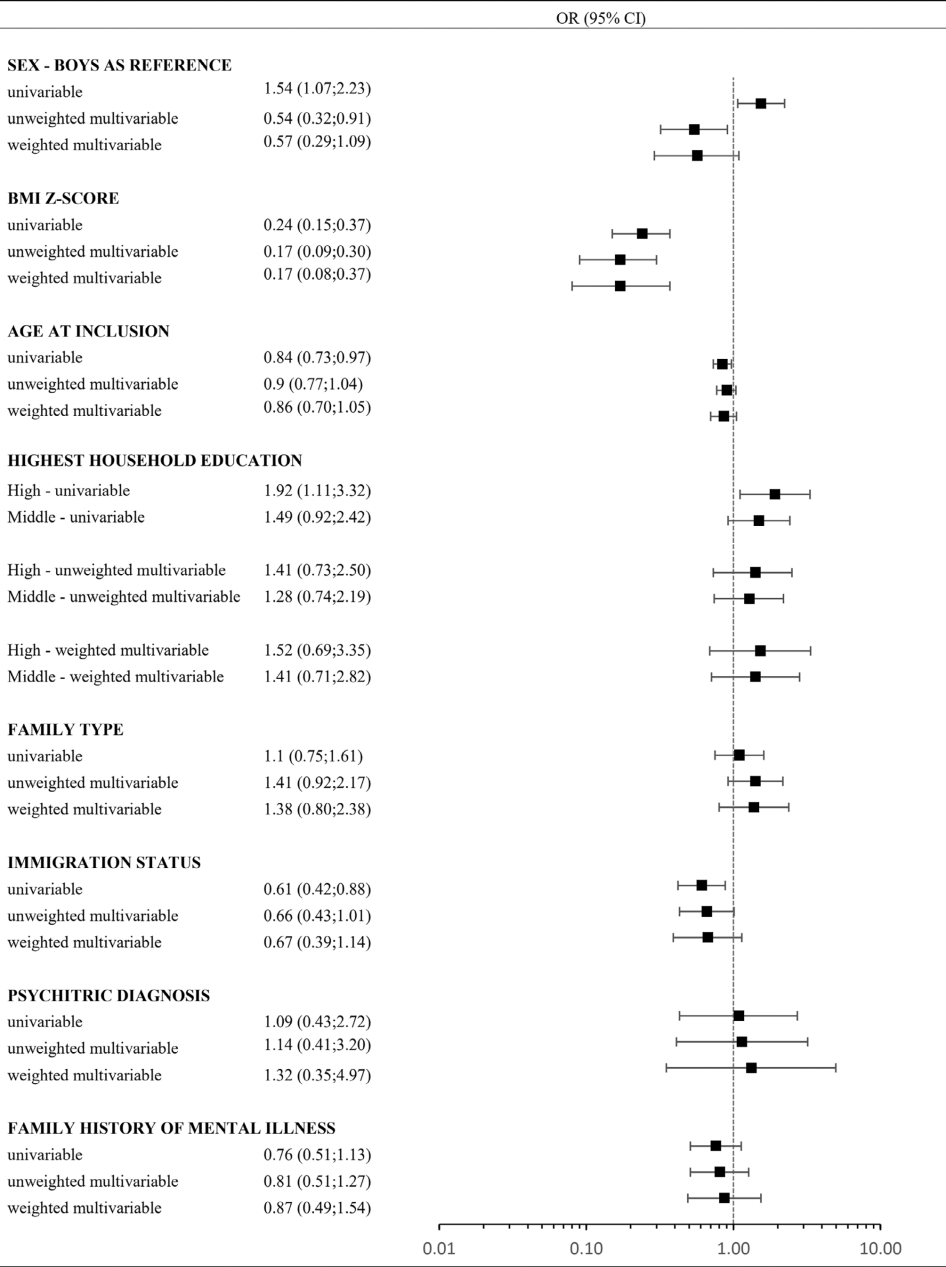
Logistic regression models, including 467 children aged 5–10 years living with obesity and a minimum of 3 years of follow‐up not receiving a lifestyle intervention, and presenting the odds ratio of being in the obesity remission group (212 children) at the end of follow‐up with the persistence of obesity group as the reference. For each co‐variable, we present univariable, unweighted multivariable, and weighted multivariable odds ratios. The included co‐variables are sex (boys as reference), BMI *z*‐score (per unit increase in SD), age (per unit increase in a year) highest completed household education (low as reference), family type (two‐adult family as reference), immigration status (Danish origin as reference), psychiatric diagnosis at the child (no diagnosis as reference) or family history of mental illness (no disposition as reference). The weighted multivariable model was weighted by the ratio of follow‐up of each individual relative to the maximum follow‐up observed in the cohort.

However, in the univariable models, increased odds of obtaining obesity remission were also observed for girls as compared to boys (OR 1.54, CI: 1.07–2.23), for high level of parental education as compared to low level (OR 1.92, CI: 1.11–3.32), for younger age (OR 0.84 per year increase, CI: 0.73–0.97) and for Danish origin (reference) as compared to immigration background (OR 0.61, CI: 0.42–0.88). In contrast to the univariable models, boys had in the unweighted multivariable model improved odds (OR 0.54, CI: 0.32–0.91) of obtaining obesity remission as compared to the girls (Figure 3).

### 3.3 Increase in BMI *z*‐score above the 80th percentile

We observed a median increase in BMI *z*‐score of 0.46 SD (Q_1_;Q_3_: 0.29; 0.64) for the 92 children (19.7%) in the highest quintile of change in BMI *z*‐score from inclusion to last observation and a decrease in BMI *z*‐score of −0.45 SD (Q_1_;Q_3_: −0.88; −0.13) for the remaining 375 children (80.3%). A mean follow‐up of 6.0 years (SD: 1.8) was observed for the children in the highest quintile, and a follow‐up of 6.3 years (SD: 1.7) for the remaining children.

At the time of inclusion and in the descriptive comparison, the group of children with the highest increase in BMI *z*‐score were significantly younger, more likely to be girls, and had a lower BMI *z*‐score at inclusion compared to the remaining children (Table 2).

**TABLE 2:** Characteristics of 467 children with obesity aged 5–10 with a minimum of 3 years of follow‐up living in Aarhus Municipality, 2010–2018 and not receiving a lifestyle intervention.

Factor	Below the 80th percentile	Above the 80th percentile
*N*	375	92
∆BMI *z*‐score (SD), median (Q_1_;Q_3_)	−0.45 (−0.88; −0.13)	0.46 (0.29; 0.64)
Sex, *n* (%)		
Boys	192 (51.2%)	36 (39.1%)
Girls	183 (48.8%)	56 (60.9%)
Age at inclusion, mean (SD)	8.1 (1.3)	7.7 (1.4)
BMI‐*z* score (inclusion), mean (SD)	3.1 (0.5)	2.8 (0.4)
Family type, *n* (%)		
Not two‐parents family	135 (36.4%)	25 (27.2%)
Two‐parents family	236 (63.6%)	67 (72.8%)
Equivalised household income (DKK), mean (SD)^a^	160 827 (1.0)	163 185 (1.0)
Highest completed education, *n* (%)		
Short	78 (21.1%)	19 (20.9%)
Middle	196 (53.0%)	47 (51.6%)
Long	96 (25.9%)	25 (27.5%)
Immigrants status, *n* (%)		
Danish	190 (51.2%)	41 (44.6%)
Immigrants	181 (48.8%)	51 (55.4%)
Family history of mental illness, *n* (%)		
No	256 (68.3%)	64 (69.6%)
Yes	119 (31.7%)	28 (30.4%)

*Note*: The children have been stratified as being above or below the 80th percentile for change in BMI *z*‐score from inclusion to end of follow‐up.

^a^ Log‐back transformed.

The odds of being in the highest quintile group (above the 80th percentile) were inversely associated with BMI *z*‐score at inclusion (0.14 per one‐unit, CI: 0.05–0.45) in the weighted multivariable model, with similar associations observed in the unweighted models.

**FIGURE 4:**
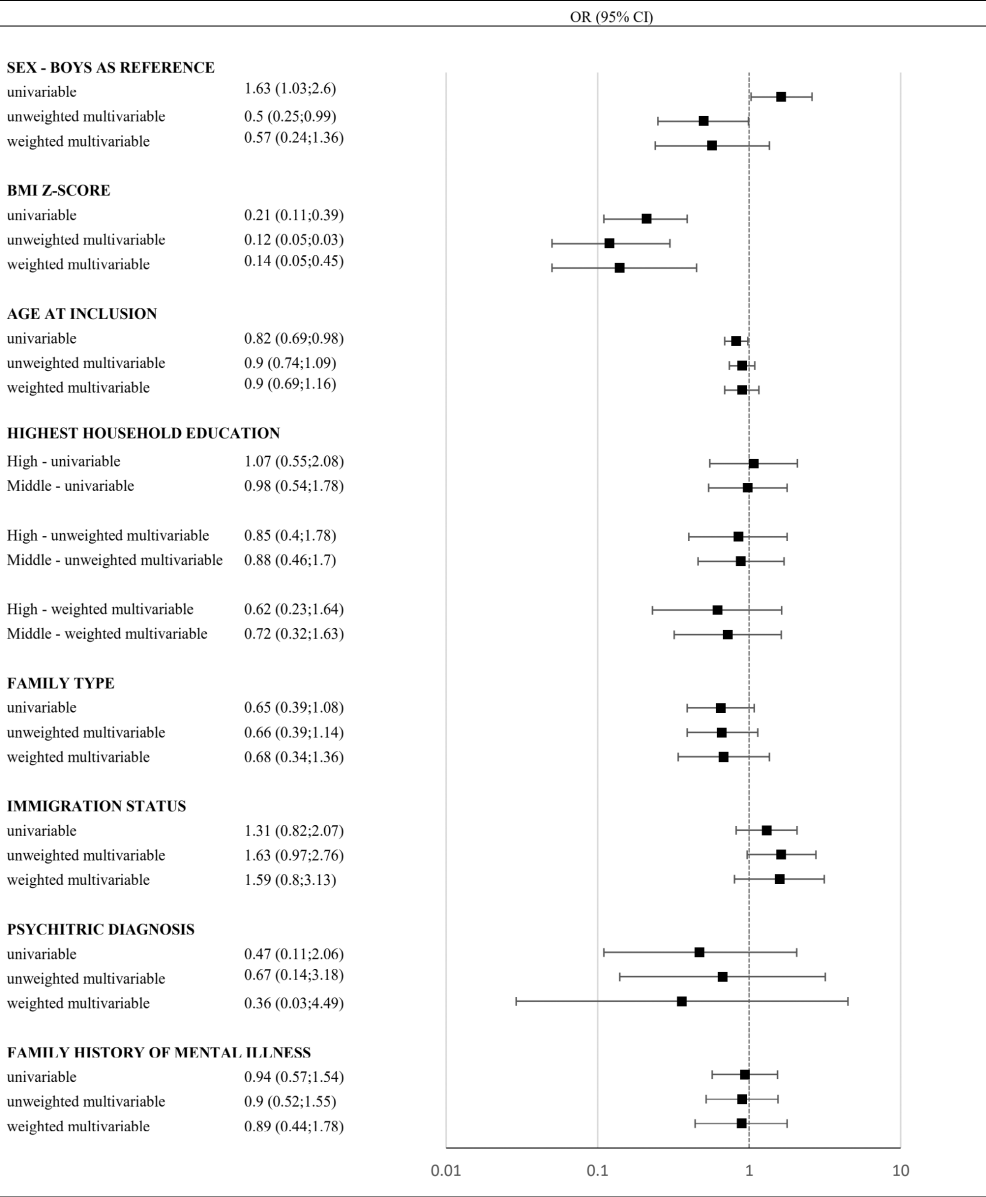
Logistic regression models, including 467 children aged 5–10 years living with obesity and a minimum of 3 years of follow‐up not receiving a lifestyle intervention, and presenting the odds ratio of being above 80th percentile for change in BMI *z*‐score with being below the 80th percentile for change in BMI *z*‐score (375 children) as reference. For each co‐variable, we present an univariable, unweighted multivariable, and weighted multivariable odds ratio. The included co‐variables are sex (boys as reference), BMI *z*‐score (per unit increase in SD), age (per unit increase in a year) highest completed household education (low as reference), family type (two‐adult family as reference), immigration status (Danish origin as reference), psychiatric diagnosis at the child (no diagnosis as reference) or family history of mental illness (no disposition as reference). The weighted multivariable model was weighted by the ratio of follow‐up of each individual relative to the maximum follow‐up observed in the cohort.

In the univariable models increased odds of being in the highest quintile group were also observed for girls as compared to boys (OR 1.63, CI: 1.03–2.6) and for children with younger age (OR 0.82 per year increase, CI: 0.69–0.98), while in the univariable models, boys had in the unweighted multivariable model higher odds of being in the highest quintile group as compared to the girls (OR 0.50, CI: 0.25–0.99) (Figure 4).

## 4 DISCUSSION

### 4.1 Findings

This study investigates the proportion and predictors of normalizing BMI and obtaining obesity remission in children already living with obesity and not receiving any lifestyle intervention.

We observed that 8% of the children normalized BMI and 45% obtained remission of obesity during a median follow‐up of more than 6 years. BMI *z*‐score at inclusion was a strong inverse predictor of normalizing BMI and for obesity remission. For our secondary, BMI *z*‐score also acted as an inverse predictor for obtaining the highest increase in BMI *z*‐score during follow‐up.

### 4.2 Comparison to previous studies

We found two studies reporting on the obesity development in children including comparable age of the children and follow‐up (~ 6 years), however, the two studies did not distinguish between children who received a lifestyle intervention and those who did not.^13^, ^14^ Wheaton et al. observed that 10% of the children with obesity normalized BMI,^14^ while obesity remission could be observed in 21.6% as reported by Luan et al.^13^ and in 29% as reported by Wheaton et al.^14^ In a study with a shorter follow‐up (i.e., 2.1 years), Foster et al. reported an obesity remission rate of 11%, while only 1% of the included children normalized BMI.^12^ An explanation for the different results as compared to our study, could be that the Danish prevalence of childhood obesity is relatively low (3%–4%) and has been stable over the past two decades,^10^ which may not be the case in other countries (e.g., the US, Australia, and Southern European countries).^13^, ^14^, ^36^ Alternative explanations could be the use of cohorts dating further back in time (e.g., 1998–1999)^13^, ^14^ and the inclusion of participants with an older age.^9^, ^12^

Furthermore, we found that BMI *z*‐score at inclusion acted as an inverse predictor for changing obesity to overweight or normal BMI and hereby support earlier findings^9^, ^16^ and we can hereby add to the literature that this association may also apply for children with obesity not receiving a lifestyle intervention.

In our secondary analyses, we observed that high baseline BMI *z*‐score seemed to be protective against the highest weight gain (being above the 80th percentile) in children already classified as having obesity. Thus, children with the highest BMI *z*‐scores at inclusion were less likely to change weight category to either overweight or overweight, and at the same time less likely to be those gaining most weight during follow‐up.

In the univariable and multivariable analyses, we observed that girls changed from having improved to impaired chances of obtaining obesity remission. Similar, but inverse, findings were observed for the odds of having a lower versus higher increase in BMI *z*‐score during follow‐up (below and above the 80th percentile). These conflicting results could be explained by a large observed difference in BMI *z*‐score between the sexes at inclusion with girls having significantly lower BMI *z*‐score at inclusion compared to the boys.

The literature is inconsistent on whether sex acts as a predictor for obesity remission and normalization of BMI in children.^13^, ^14^, ^16^, ^36^ Some studies report that boys are more likely than girls to obtain obesity remission,^13^, ^36^ while other studies report that girls are more likely than boys to normalize BMI.^13^, ^14^ A explanation could be a different study design and setting, using univariable logistic regressions, or adjusting only for a few variables, instead of including all relevant variables in a multivariable model, hereby making the authors less likely to remove potential confounding.^13^, ^16^

Younger children have better chances for obesity remission and for normalizing weight^12^, ^15^, ^16^, ^17^, ^36^, ^37^; however, there is disagreement about the exact window of opportunity and different ages have been suggested as the optimal moment to intervene. Kim et al. reported that children aged 5–6 years have a better chance of remission,^17^ while McGinty et al. observed that weight will increase with a faster rate between ages 12–14 in children with severe obesity.^16^ Following children with obesity, Whitaker et al. observed a substantially increased OR of 17.5 for persistence of obesity in early adulthood among children with obesity aged 10–14, while lower OR of 1.7 was found in children aged 0.6–3 years.^15^ In this study, we did not observe significant associations between age and change in weight status; however, we did observe non‐significant tendency between age and obesity remission suggesting a weaker association. A possible explanation could be the study's inclusion criteria, focusing only on children of a certain age (aged 5–10), and hereby not making our result generalizable to older children.

An inverse association between SES and the chance of obtaining obesity remission has been reported for children, with remission rates of 32.7% for children in the highest SES strata as compared to 16.7% in the lowest.^13^ These findings were supported by Wheaton et al.,^14^ who observed an increased risk of persistence of obesity in children with low SES, and Chen et al., reporting higher rates of persistence of obesity in children from low SES schools.^36^ In our study, we only observed subtle trends suggesting weaker associations between lower SES and persistence of obesity as well as the risk of increasing BMI *z*‐score. An explanation for these non‐significant results could be the relatively small number of included children, differences in the SES classifications, and that Danish citizens (children) are provided with free education, child support, and health care, which may result in relatively smaller differences across the societal strata and less economic inequality as compared to other countries.^13^, ^14^, ^36^

In this study, we observe non‐significant trends concerning immigrant background. This may be due to the categorization of immigration status into only two strata. Immigration status represents a very heterogeneous group of children with mixed SES, ethnicity, integration in the Danish society, and differing abilities to follow the recommendations of the Danish dietary and exercise recommendations. A more nuanced stratification could potentially have altered the results. That could also explain, why some reports observe significant associations,^19^, ^36^ while others do not.^12^, ^16^ The group of children with immigrant backgrounds was in this study too small for being subdivided any further, however, it could be interesting to explore this in future studies.

### 4.3 Strengths and limitations of this study

A clear strength of this study was the long follow‐up with a median of more than 6 years. Furthermore, by excluding children with obesity who accepted or who refused to participate in the intervention, it became possible to follow weight development in children. By combining existing data from mandatory health check‐ups with variables from the national registries, we were able to reduce the risk of selection and recall bias as well as differential loss to follow‐up.^21^ By using a multivariable analysis, it became possible to investigate several predictors in the same model and hereby remove potential confounding.

However, the study also had limitations. We only included a relatively small sample size (467 children) of whom only 37 children normalized BMI, and due to the limitation of including one co‐variable for every 10 observations in the strata with the fewest cases, we therefore could not account for factors like SES, family type, immigration status, and psychiatric disorders. This a priori decision on number of co‐variables used was made to preserve precision of the estimates. Information on sedentary behaviour, maternal and paternal weight, genetic factors, birth weight of the child, consumption of sugar‐sweetened beverages, vegetable consumption, social well‐being, and interpersonal skills were not available using the accessible data, and including such co‐variables could potentially have improved the model.^14^, ^15^, ^18^, ^19^ Due to the available data, we had to choose a pragmatic design, in which we only included the latest observation for each child, a design that made us unable to describe weight fluctuations, which has been reported in the literature.^9^, ^13^ Furthermore, we believe that weighting the model by the ratio of follow‐up of each individual relative to the maximum follow‐up was the best way to account for the individual differences in follow‐up. Danish children are assessed regularly during childhood by school health nurses and general practitioners. These clinical assessments are not comparable to an intervention, even though they may have some preventive effects. This could limit the generalization of our results, particularly when comparing them to countries including different systems. The children could have received an alternative treatment for obesity. However, during this period, Aarhus Municipality did not provide alternative treatments for childhood obesity, therefore, the only other options available for the families would have been to attempt weight loss on their own, seek the assistance from general practitioners, or apply for enrolment in the multi‐component lifestyle camps.^38^ We did not have any information on children attending these lifestyle camps, however, the camps only have the capacity to include a limited number of participants, and the effect on BMI may be only temporary.^39^

## 5 CONCLUSION

In this cohort of Danish children with obesity not invited to participate in the available lifestyle intervention, 8% of the children normalized BMI, while 45% experienced remission of obesity during a median follow‐up of more than 6 years.

We found that children with the highest BMI *z*‐scores at inclusion were less likely to change weight category to either overweight or obesity, and at the same time less likely to be among those gaining most weight during follow‐up compared to children with lower BMI *z*‐scores at inclusion. This suggests that some children with obesity are more likely to achieve a presumably healthier weight and BMI *z*‐score without an intervention.

By allocating resources from this group to the children less fortunate, we could potentially reduce unnecessary stigmatization for the children, however, more research is needed to explore this association further.

## FUNDING INFORMATION

Rasmus Møller Jørgensen is supported by an unrestricted public grant by the Danish Regions, Denmark “The Joint Grant for Prevention” (grant number EMN‐2019‐00852 1431585). Iris Iglesia was founded by RICORS funded by the Recovery, Transformation and Resilience Plan 2017–2020, ISCIII, and by the European Union–Next Generation EU, ref. RD21/0012/0012. Jens Meldgaard Bruun, Henrik Støvring, and Jane Nautrup Østergaard are all employed at Steno Diabetes Center Aarhus and partly funded by an unrestricted grant from the Novo Nordisk Foundation. None of the above‐mentioned foundations had any role in the design or conduction of this study.

## CONFLICT OF INTEREST STATEMENT

All authors (except for IIA and LM) are employed at Steno Diabetes Center Aarhus, Aarhus University Hospital, a public hospital and research institution situated in the Central Denmark Region, which is partly funded by an unrestricted grant from the Novo Nordisk Foundation.

## Data Availability

Anonymized data can be shared upon publication to researchers on reasonable request. Data from the national registries and STIL cannot be shared due to the Danish General Data Protection Regulation.
